# Computerized texture analysis of pulmonary nodules in pediatric patients with osteosarcoma: Differentiation of pulmonary metastases from non-metastatic nodules

**DOI:** 10.1371/journal.pone.0211969

**Published:** 2019-02-08

**Authors:** Yeon Jin Cho, Woo Sun Kim, Young Hun Choi, Ji Young Ha, SeungHyun Lee, Sang Joon Park, Jung-Eun Cheon, Hyoung Jin Kang, Hee Young Shin, In-One Kim

**Affiliations:** 1 Department of Radiology, Seoul National University Hospital, Jongno-gu, Seoul, Republic of Korea; 2 Department of Radiology, Seoul National University College of Medicine, Jongno-gu, Seoul, Republic of Korea; 3 Institute of Radiation Medicine, Seoul National University Medical Research Center, Jongno-gu, Seoul, Republic of Korea; 4 Department of Radiology, Gyeongsang National University Changwon Hospital, Changwon, Republic of Korea; 5 Cancer Research Institute, Seoul National University, Seoul, South Korea; 6 Department of Pediatrics, Seoul National University Hospital, Jongno-gu, Seoul, Republic of Korea; 7 Department of Pediatrics, Seoul National University College of Medicine, Jongno-gu, Seoul, Republic of Korea; Universite de Nantes, FRANCE

## Abstract

**Objective:**

To retrospectively evaluate the value of computerized 3D texture analysis for differentiating pulmonary metastases from non-metastatic lesions in pediatric patients with osteosarcoma.

**Materials and methods:**

This retrospective study was approved by the institutional review board. The study comprised 42 pathologically confirmed pulmonary nodules in 16 children with osteosarcoma who had undergone preoperative computed tomography between January 2009 and December 2014. Texture analysis was performed using an in-house program. Multivariate logistic regression analysis was performed to identify factors for differentiating metastatic nodules from non-metastases. A subgroup analysis was performed to identify differentiating parameters in small non-calcified pulmonary nodules. The receiver operator characteristic curve was created to evaluate the discriminating performance of the established model.

**Results:**

There were 24 metastatic and 18 non-metastatic lesions. Multivariate analysis revealed that higher mean attenuation (adjusted odds ratio [OR], 1.014, P = 0.003) and larger effective diameter (OR, 1.745, P = 0.012) were significant differentiators. The analysis with small non-calcified pulmonary nodules (7 metastases and 18 non-metastases) revealed significant inter-group differences in various parameters. Logistic regression analysis revealed that higher mean attenuation (OR, 1.007, P = 0.008) was a significant predictor of non-calcified pulmonary metastases. The established logistic regression model of subgroups showed excellent discriminating performance in the ROC analysis (area under the curve, 0.865).

**Conclusion:**

Pulmonary metastases from osteosarcoma could be differentiated from non-metastases by using computerized texture analysis. Higher mean attenuation and larger diameter were significant predictors for pulmonary metastases, while higher mean attenuation was a significant predictor for small non-calcified pulmonary metastases.

## Introduction

Osteosarcoma is the most common primary malignant bone tumor in children and adolescents [1]. Pulmonary metastasis is the most common metastatic disease associated with osteosarcoma, and approximately 15–20% of the patients have metastatic lesions when they are first diagnosed with osteosarcoma [1, 2]. Furthermore, pulmonary metastasis is a major prognostic factor for survival. The 5-year survival rate of patients with pulmonary metastasis has been reported to drop to 37%, whereas the 5-year survival rate of patients without metastatic disease is approximately 60–70% [3, 4]. Resection of the pulmonary metastases is known to be associated with improved survival in patients with osteosarcoma and resectable lung metastases [4]. Therefore, the identification and differentiation of pulmonary metastases from non-metastatic lesions is very important for appropriate treatment. Computed tomography (CT) is the most widely used imaging technique for detecting and identifying pulmonary nodules. However, distinguishing pulmonary metastasis from benign pulmonary nodules on CT is difficult, and only 64–74% of the nodules could be correctly identified by experienced radiologists via subjective assessment of CT findings [5].

In recent years, increasing effort has been put into the quantitative evaluation of CT images. A number of studies have shown that computerized texture analysis could be a promising method for lesion identification and differentiation [6]. Osteosarcoma is pathologically quite different from benign pulmonary lesions in that it contains an osteoid matrix. Therefore, quantitative image analysis could provide additional information for differentiating pulmonary metastases on CT. The purpose of this study was to retrospectively investigate the value of computerized 3D image analysis for differentiating pulmonary metastases from non-metastatic nodules in pediatric patients with osteosarcoma.

## Materials and methods

This study was approved by the Institutional Review Board (IRB) of Seoul National University Hospital (Seoul, Republic of Korea). The approval number is 1706-060-859. We were given exemption from getting informed consents by the IRB. It was a retrospective study. And personal identifiers were all removed and the data were anonymously analyzed.

### Study population

By reviewing the surgical and pathologic databases of our institute, we identified 21 children with pathologically confirmed osteosarcoma who underwent metastasectomy 31 times in total between January 1, 2009 and December 31, 2014. Among them, only 16 children underwent preoperative non-contrast CT examinations with a section thickness of 1 mm. The surgical findings documented whether surgically resected nodules matched the location of the nodules found on CT. In total, 86 nodules were resected via surgery from these 16 children. Among the 86 pulmonary nodules, 44 were excluded from our study because we were not able to identify the corresponding nodules on CT because of insufficient surgical reports. Finally, 16 patients (mean age, 13.8 years; age range, 7–18 years; 10 boys and 6 girls) with 42 pathologically proven pulmonary nodules were included in our study. Among the 42 pulmonary nodules, 24 were pulmonary metastases and 18 were non-metastatic pulmonary nodules (12 intrapulmonary lymph nodes, 4 non-neoplastic lung parenchyma, 1 organizing pneumonia, and 1 atypical adenomatous hyperplasia).

**Fig 1:**
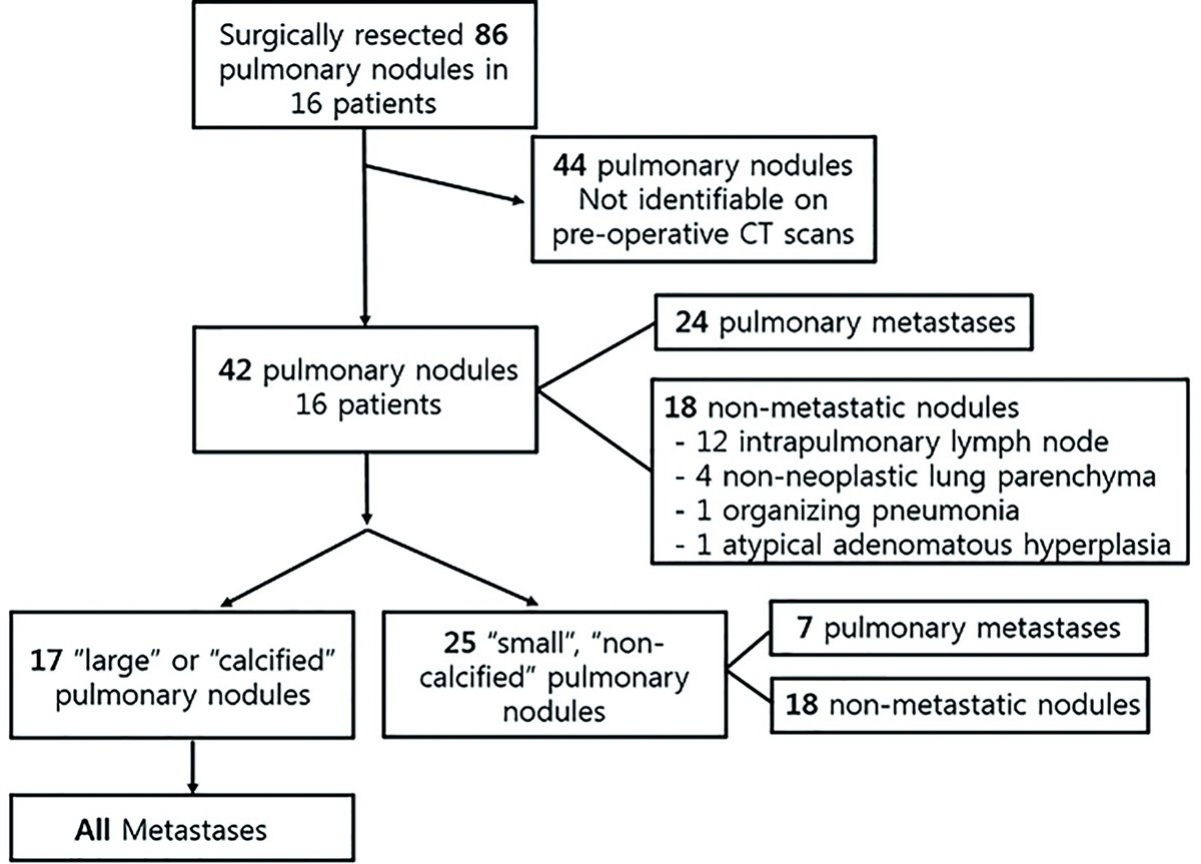
Flow diagram illustrating the inclusion/exclusion process of this study.

Subgroup analysis was performed to identify significant predictors of small pulmonary metastases among the small non-calcified pulmonary nodules. A small pulmonary nodule was defined as a nodule having a size of 5 mm or smaller. We determined the nodule size by using the effective diameter extracted from the texture analysis software. The presence of calcification was determined by visual inspection on the mediastinal window setting CT images (window width, 400 Hounsfield units [HU]; window level, 30 HU). For exact identification of the presence of calcification, we used magnification tools provided in the texture analysis software. In total, 25 small non-calcified pulmonary nodules were eligible for subgroup analysis. Among the 25 pulmonary nodules, 7 were pulmonary metastases and 18 were confirmed as non-neoplastic lesions (Fig 1).

### CT imaging

The CT scan acquired just prior to pulmonary metastasectomy was evaluated. All CT examinations were conducted without intravenous contrast material injection by using one of five available CT scanners: Sensation-16, Somatom Definition FLASH (Siemens Medical Systems, Erlangen, Germany), Brilliance-64, Ingenuity (Philips Medical Systems, Best, The Netherlands), and Aquilion One (Toshiba Medical Systems, Otawara, Japan). All chest CT scans were performed using dose modulation with the following scanning parameters: tube voltage, 80–120 kVp; tube current-time products, 20–132 mAs; reconstruction slice thickness, 1.0 mm; slice interval, 1.0 mm; and a medium-sharp reconstruction algorithm. The mean interval between the preoperative CT and surgery was 17.2 ± 11.5 days (range, 1–35 days). The detection and annotation of pulmonary nodules were performed by experienced pulmonary radiologist (30 years-experienced chest radiologist) with assistance of Computer-aided diagnosis (CAD)

### Computerized texture analysis

**Fig 2:**
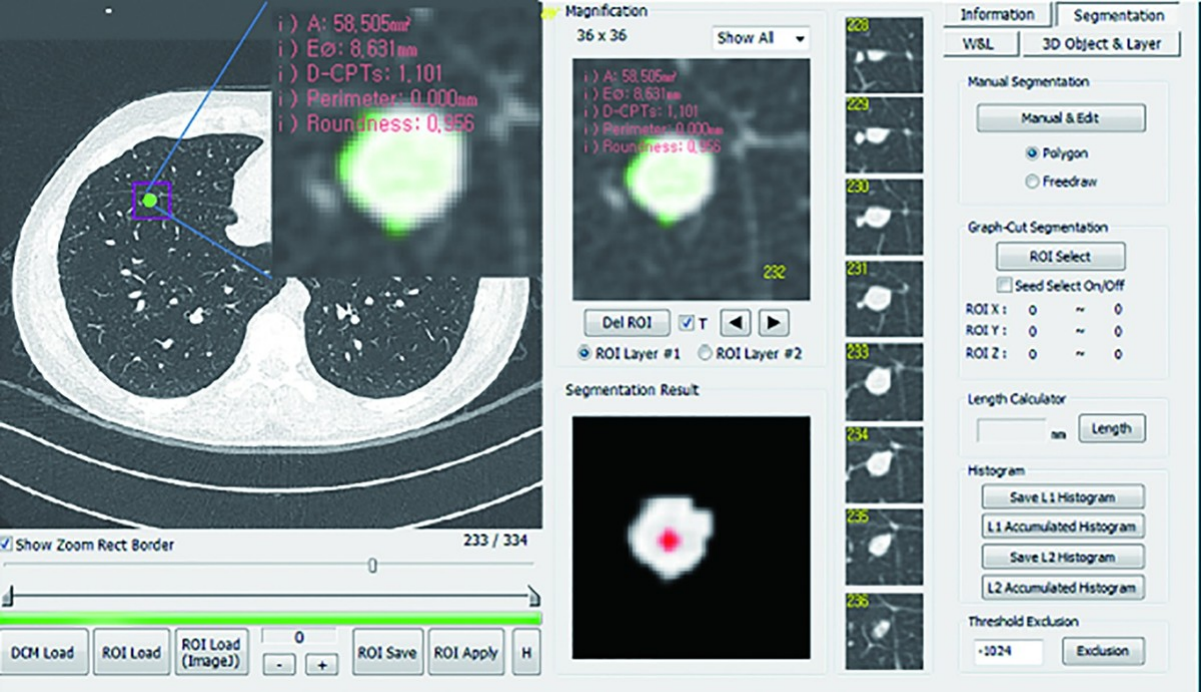
Texture analysis process. The screen-capture image shows the brief process of texture analysis using the in-house software program. The segmentation of pulmonary nodules is manually performed, and texture features of the nodules are automatically extracted by the software program.

An in-house software program (Medical Imaging Solution for Segmentation and Texture Analysis [MISSTA]), which was coded in the C++ language with Microsoft Foundation Classes (Microsoft Corporation, Redmond, WA) was used for the segmentation of pulmonary nodules and automated quantification of morphologic and textural parameters. Manual segmentation of the pulmonary lesions was conducted by one radiologist (Y. J. C., with 3 years of experience in pediatric radiology) under the supervision of an experienced radiologist (Y. H. C., with 12 years of experience in pediatric radiology) in the lung window setting (window width, 1500 HU; window level, -700 HU). Regions of interest were drawn along the boundary of each pulmonary nodule on all image sections of each pulmonary nodule. To exclude the surrounding normal lung parenchyma, any pixels with attenuation of less than -500 HU were automatically discarded. An example of manual segmentation performed using the MISSTA software is presented in Fig 2. After manual segmentation of the nodules, their texture features were automatically calculated and extracted. The analyzed CT texture features included first-order features, second-order texture features, and morphologic features [7, 8]. First-order statistics included mean attenuation, standard deviation, variance, skewness, and kurtosis. Second-order statistics were obtained using the gray-level co-occurrence matrix (GLCM) and included GLCM moments, angular second moment (ASM), inverse difference moment (IDM), contrast, and entropy. The morphologic features recorded included the effective diameter, surface area, volume, sphericity, and discrete compactness. The effective diameter was defined as the diameter of a sphere whose volume is equal to the segmented nodule. Please refer to the Supporting Information (S1 Appendix) for detailed information on the texture features.

### Reproducibility of texture features

The inter-observer reproducibility of the measurements was assessed by two radiology specialists. Two radiologists (12 year experience; Y.H.C. and 6 year experience; Y. J. C.) performed manual segmentation of the pulmonary nodules independently with anonymized image data.

### Comparison with conventional assessment of pulmonary nodules

To compare the differentiating performance of texture features to that of conventional CT characteristics, one radiologist (12 year experience; Y.H.C.) performed measurement of long diameter, mean attenuation and standard deviation of attenuation in each pulmonary nodule. The “conventional long diameter” of each pulmonary nodules was determined in the representative section where each pulmonary nodules were best identified. The “conventional mean attenuation” of each pulmonary nodules was also determined in the representative section of lesions, the attenuation was measured in the picture archiving and communication system (PACS) with circular region of interest.

### Statistical analysis

All data were analyzed by using IBM SPSS Statistics for Windows/Macintosh, Version 21.0 (IBM Corp., Armonk, NY) and MedCalc version 12.7 (MedCalc Software, Ostend, Belgium). P values less than .05 were considered significant. We evaluated the inter-observer reproducibility for each texture features using the intraclass correlation coefficient (ICC) with 95% confidence intervals (CIs). An ICC above 0.90 indicates excellent reproducibility, an ICC between 0.75 and 0.90 indicates good reproducibility, an ICC between 0.50 and 0.75 indicates moderate reproducibility and an ICC below 0.50 indicates poor reproducibility. To compare first-order statistics, second-order statistics, and morphologic features between pulmonary metastases and non-metastatic pulmonary lesions in children with osteosarcoma, the independent sample *t*-test was applied. Thereafter, logistic regression analysis using the backward-elimination method with texture parameters shown to be of statistical significance in the univariate analysis was performed to identify significant independent predictors for differentiating pulmonary metastases from non-metastatic pulmonary lesions. Receiver operator characteristic (ROC) curve was constructed to determine the optimal cutoff values for statistically significant variables. Subgroup analyses were also performed in the same manner as the total group analyses. To compare the differentiating performance between using texture features and conventional CT characteristics, ROC comparison was performed with significant predictors for pulmonary metastases.

## Results

### Patients

The majority of patients (9 of 16 [56%]) had osteoblastic-type osteosarcoma. The other histologic subtypes included the chondroblastic type (2 of 16 [13%]), fibroblastic type (1 of 16 [6%]), and giant-cell-rich type (1 of 16 [6%]). The primary sites of primary osteosarcoma were the femur, tibia, and humerus. The histologic subtype could not be determined using surgical specimens because of the absence of viable tumors after neoadjuvant chemotherapy. All patients underwent neoadjuvant and postoperative chemotherapy using a variety of therapeutic agents, including doxorubicin, cisplatin, high-dose methotrexate, ifosfamide, and etoposide.

### Texture features

The intra-observer reproducibility of texture features is presented in Table 1. All the ICCs except ICC of discrete compactness showed excellent reproducibility (> 0.9) between two investigators. The ICC of discrete compactness showed good reproducibility (0.821)

**Table 1:** Intraclass correlation coefficients for inter-observer reproducibility of texture features.

Characteristic	Observer 1	Observer 2	ICC (95% CIs)
Mean attenuation (HU)	-72.1 ± 213.2	-76.9 ± 217.3	0.996 (0.993–0.998)
Standard deviation (HU)	225.4 ± 125.8	222.2 ± 127.5	0.993 (0.987–0.996)
Variance (HU)	66248.8 ± 92125.6	65252.8 ± 95591.1	0.997 (0.994–0.998)
Skewness	0.404 ± 0.546	0.081 ± 0.520	0.929 (0.869–0.962)
Kurtosis	-0.351 ± 0.886	-0.389 ± 0.879	0.975 (0.954–0.987)
Effective diameter (mm)	6.7 ± 9.3	6.6 ± 9.2	0.999 (0.999–1.000)
Surface area (mm^2^)	251.6 ± 496.7	248.2 ± 506.2	0.998 (0.997–0.999)
Volume (mm^3^)	409.7 ± 1186.6	398.7 ± 1172.0	1.000 (0.999–1.000)
Sphericity	0.800 ± 0.138	0.791 ± 0.128	0.950 (0.907–0.973)
Discrete compactness	-0.364 ± 0.832	-0.488 ± 0.879	0.821 (0.669–0.903)
GLCM moments	1.9 ± 0.3	1.9 ± 0.3	0.946 (0.895–0.972)
GLCM ASM	0.004 ± 0.005	0.004 ± 0.005	0.992 (0.985–0.996)
GLCM IDM	0.005 ± 0.003	0.005 ± 0.004	0.989 (0.979–0.994)
GLCM contrast	109554.5 ± 92995.4	106283.8 ± 94857.2	0.994 (0.988–0.997)
GLCM entropy	2.9 ± 0.8	2.9 ± 0.8	0.999 (0.998–0.999)

ICC = intraclass correlation coefficient; CIs = confidence intervals; HU = hounsfield unit

Note. Except where indicated, data are mean ± standard deviation.

Tables 2 and 3 show the summary statistics of the extracted CT features in pulmonary metastases and non-metastatic pulmonary nodules. In first-order statistics, pulmonary metastases had higher mean attenuation and larger standard deviation and variance than did the non-metastatic pulmonary lesions. Second-order statistics, including GLCM ASM, IDM, and entropy, yielded statistically significant results. Pulmonary metastases were significantly larger than non-metastatic lesions in terms of effective diameter, surface area, and volume. Pulmonary metastases also showed higher discrete compactness than did non-metastatic lesions. However, only higher mean attenuation and negative skewness showed statistical significance in the subgroup analysis with small non-calcified pulmonary nodules.

**Table 2:** Histographic, Volumetric, and morphologic features of pulmonary metastases and non-metastatic lesions in all the pediatric patients with osteosarcoma.

Characteristic	Pulmonary metastases (n = 24)	Non-metastatic lesion (n = 18)	P-value*
Mean attenuation (HU)	51.4 ± 198.6	-242.4 ± 75.7	<0.001
Standard deviation (HU)	272.0 ± 146.9	159.6 ± 39.6	0.001
Variance (HU)	94666.4 ± 116166.8	27196.7 ± 13103.8	0.009
Skewness	-0.069 ± 0.488	0.2334 ± 0.512	0.059
Kurtosis	-0.214 ± 0.924	-0.578 ± 0.773	0.184
Effective diameter (mm)	10.1 ± 11.1	2.0 ± 0.9	0.002
Surface area (mm^2^)	410.0 ± 620.1	36.4 ± 26.2	0.007
Volume (mm^3^)	695.6 ± 1507.2	15.8 ± 14.6	0.037
Sphericity	0.777 ± 0.151	0.821 ± 0.091	0.247
Discrete compactness	-0.003 ± 0.558	-0.990 ± 0.699	<0.001
GLCM moments	1.9 ± 0.2	1.8 ± 0.3	0.058
GLCM ASM	0.001 ± 0.003	0.007 ± 0.006	0.004
GLCM IDM	0.006 ± 0.003	0.003 ± 0.003	0.015
GLCM contrast	124301.1 ± 121736.8	86076.5 ± 14546.1	0.141
GLCM entropy	3.4 ± 0.8	2.3 ± 0.4	<0.001

Note. Except where indicated, data are mean ± standard deviation. GLCM = gray-level co-occurrence matrix, ASM = angular second moment, IDM = inverse difference moment.

* Independent-sample *t*-test.

**Table 3:** Histographic, Volumetric, and morphologic features of pulmonary metastases and non-metastatic lesions in small non-calcified pulmonary nodules.

Variable	Pulmonary metastases (n = 7)	Non-metastatic lesions (n = 18)	P-value*
Mean attenuation (HU)	-116.5 ± 91.8	-242.4 ± 75.7	0.002
Standard deviation (HU)	195.6 ± 40.0	159.6 ± 39.6	0.054
Variance (HU)	39658.2 ± 15900.2	27196.7 ± 13103.8	0.056
Skewness	-0.191 ± 0.252	0.233 ± 0.512	0.049
Kurtosis	-1.031 ± 0.213	-0.578 ± 0.773	0.144
Effective diameter (mm)	3.4 ± 1.7	2.0 ± 0.9	0.080
Surface area (mm^2^)	75.5 ± 49.5	36.4 ± 26.2	0.086
Volume (mm^3^)	45.0 ± 35.9	15.8 ± 14.6	0.077
Sphericity	0.863 ± 0.091	0.821 ± 0.091	0.313
Discrete compactness	-0.403 ± 0.716	-0.990 ± 0.699	0.074
GLCM moments	1.9 ± 0.3	1.8 ± 0.3	0.249
GLCM ASM	0.004 ± 0.005	0.007 ± 0.006	0.361
GLCM IDM	0.005± 0.005	0.003 ± 0.003	0.227
GLCM contrast	96101.5 ± 44711.6	86076.5 ± 14546.1	0.581
GLCM entropy	2.6 ± 0.5	2.3 ± 0.4	0.104

Note. Except where indicated, data are mean ± standard deviation. GLCM = gray-level co-occurrence matrix, ASM = angular second moment, IDM = inverse difference moment.

* Independent-sample *t*-test.

### Logistic regression analysis

**Fig 3:**
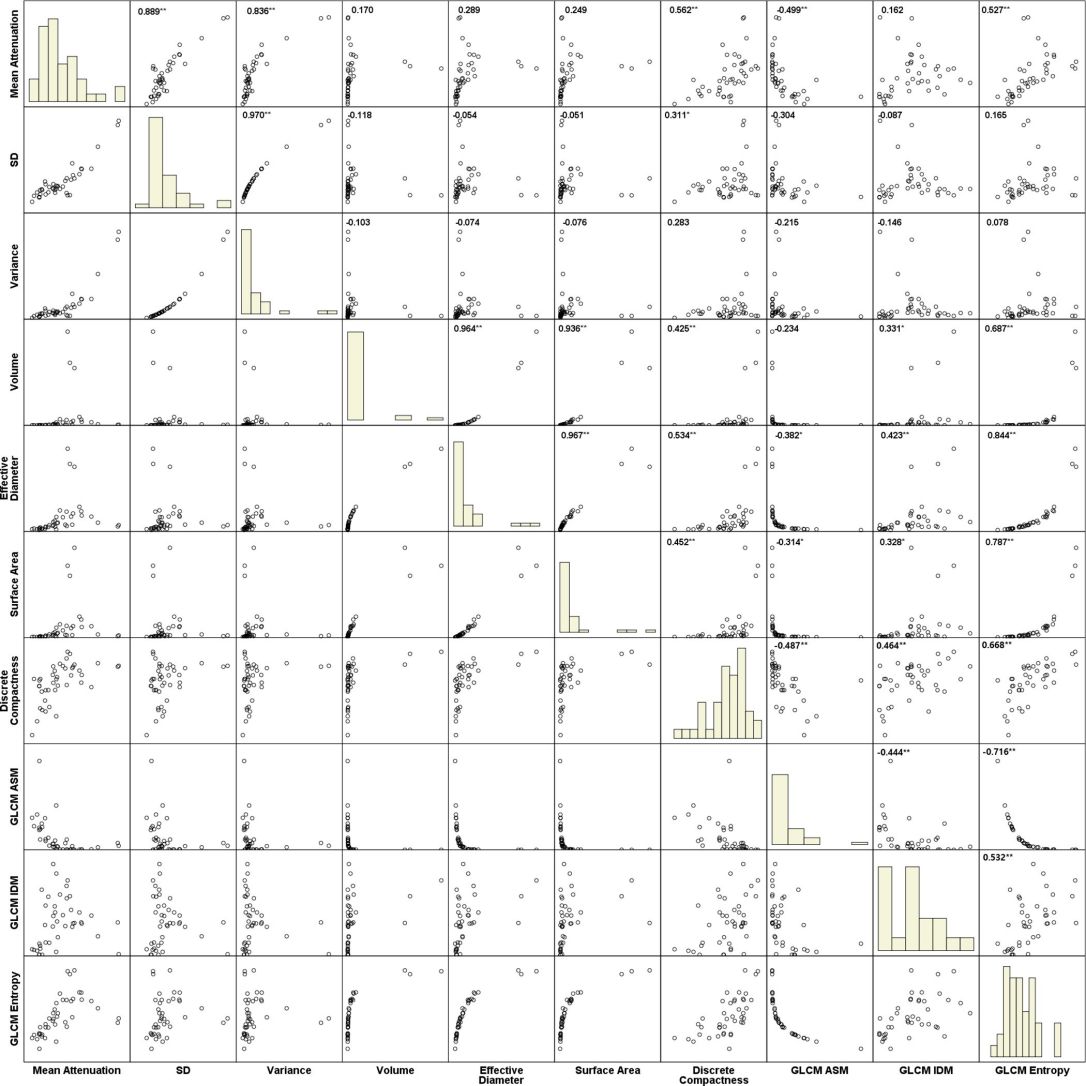
Correlation matrix of texture features. The correlation matrix was created with 10 of 15 texture features those showed statistically significant differences in independent t-test. The standard deviation and variance showed significant correlation with mean attenuation. The surface area, volume and GLCM entropy showed significant correlation with effective diameter. Pearson correlation coefficient of each pair of texture features was shown in the upper right corner of the scatter plot. *P < 0.05; **P < 0.01.

**Fig 4:**
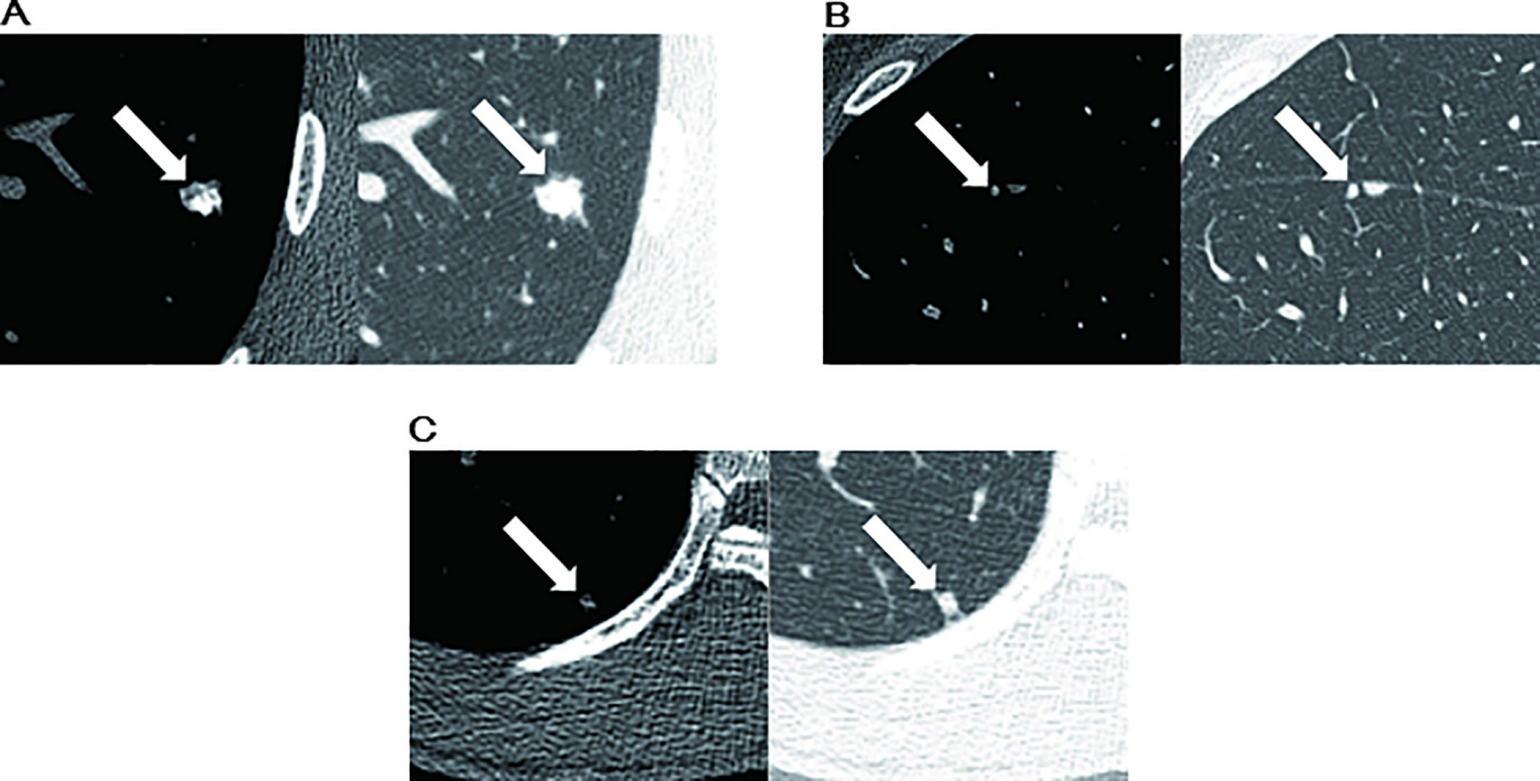
Texture analysis of pulmonary nodules: Metastatic nodules versus non-metastatic nodules. (A) CT scan shows a 12.8-mm solid pulmonary nodule (arrow) with calcification. This nodule shows high mean attenuation (123.6 ± 289.6 HU). The pulmonary nodule was confirmed as a pulmonary metastasis. (B) CT scan shows a 2.9-mm small non-calcified pulmonary nodule (arrow). This nodule shows relatively high mean attenuation (-8.8 ± 255.8 HU). It was confirmed as a pulmonary metastasis. (C) CT scan shows a 2.4-mm small non-calcified pulmonary nodule (arrow). This nodule has relatively low mean attenuation (-229.9 ± 212.3 HU). It was confirmed as an intrapulmonary lymph node.

Logistic regression analysis was performed using texture parameters shown to be of statistical significance in the univariate analysis; these included mean attenuation, standard deviation, effective diameter, discrete compactness, and GLCM ASM, GLCM IDM, and GLCM entropy. Among the statistically significant variables from the independent-sample *t*-test, significant correlation was demonstrated between mean attenuation, standard deviation, variance and effective diameter, surface area, volume and GLCM entropy (Pearson correlation coefficient > 0.8). In regression analysis, to avoid multicollinearity, standard deviation, variance, surface area, volume and GLCM entropy were excluded from the multivariate analysis (Fig 3). The variance inflation factors (VIF) of five variables those were mean attenuation, effective diameter, discrete compactness, GLCM_ASM and GLCM IDM were less than 5. Logistic analysis also revealed that higher mean attenuation (adjusted odds ratio, 1.014 [95% confidence interval, 1.005–1.024]; P = .003) and larger effective diameter (adjusted odds ratio, 1.745 [95% confidence interval, 1.129–2.698]; P = .012) were significant differentiators for pulmonary metastases (Table 4). For small non-calcified pulmonary nodules, higher mean attenuation (adjusted odds ratio, 1.007 [95% confidence interval, 1.002–1.012]; P = .008) was the only predictor of pulmonary metastases (Table 4). Representative cases are shown in Fig 4A, 4B and 4C.

**Table 4:** Results of logistic regression analysis for predictors of pulmonary metastases and non-metastatic lesions in pulmonary nodules.

	Variable	Adjusted Odds Ratio	P-value*
Total Group	Mean attenuation (HU)	1.014 (1.005–1.024)	0.003
	Effective diameter (mm)	1.745 (1.129–2.698)	0.012
Non-calcified Small Nodules	Mean attenuation (HU)	1.007 (1.002–1.012)	0.008

Note. Data are adjusted odds ratios per one standard deviation change; data in parentheses are 95% confidence intervals.

### ROC curve

**Fig 5:**
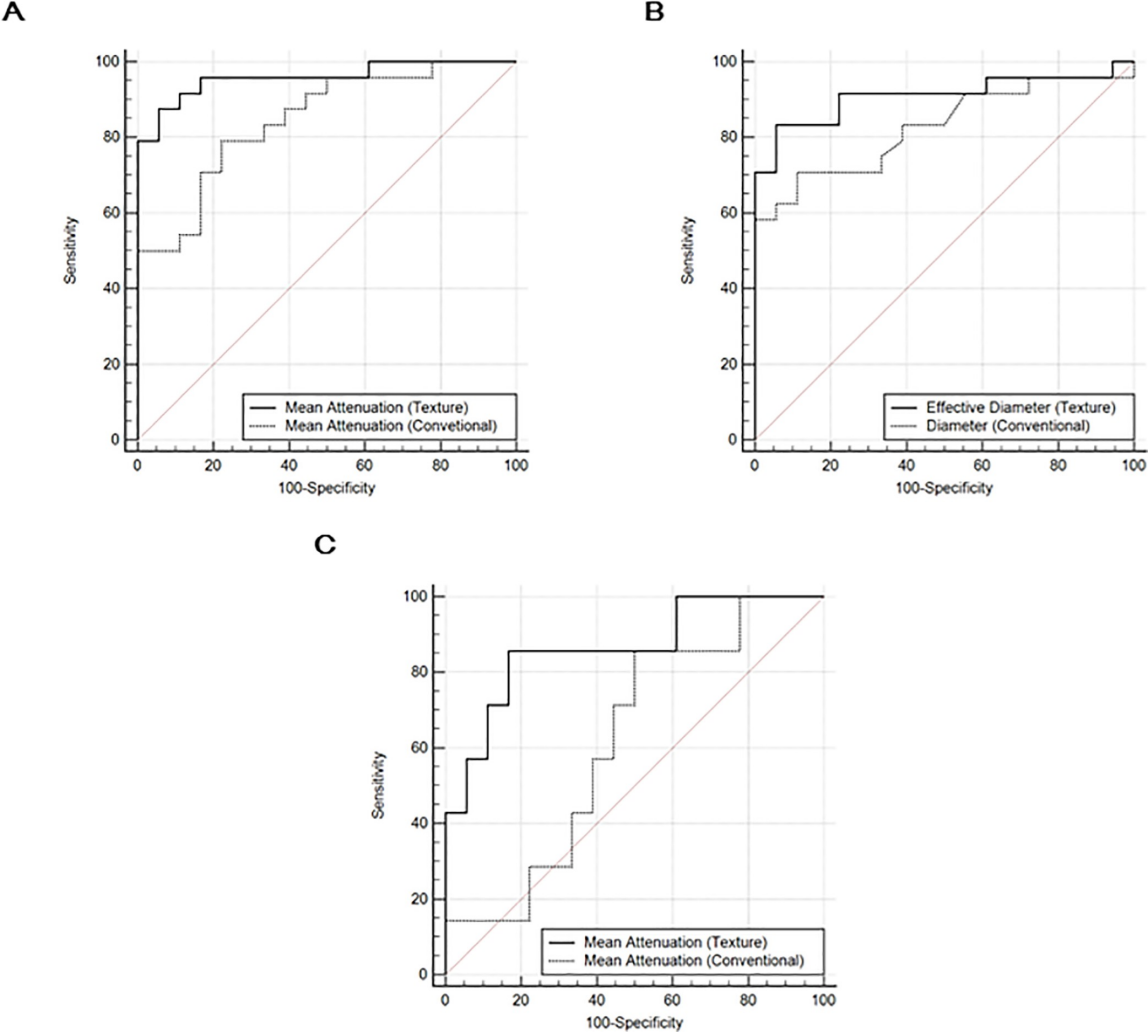
Receiver operator characteristic (ROC) curve analysis. Receiver operator characteristic (ROC) curve for mean attenuation and effective diameter obtained by texture analysis and conventional measurement for differentiating pulmonary metastases and non-pulmonary metastatic lesions. ROC was performed for mean attenuation (A) and effective diameter (B) in the total group, and for mean attenuation in the small non-calcified nodule group (C).

When we constructed the ROC curves, the optimal threshold value for mean attenuation was -153.5HU with 95.8% sensitivity and 83.3% specificity, and the optimal threshold value for effective diameter was 3.3 mm with 83.3% sensitivity and 94.4% specificity. The area under the curve (AUC) was 0.958 (95% CIs, 0.847–0.996, P < .001) and 0.910 (95% CIs, 0.780–0.976, P < .001) for mean attenuation and effective diameter, respectively (Fig 5A and 5B).

In the subgroup analysis, the optimal threshold value for mean attenuation was -153.5 HU with 85.7% sensitivity and 83.3% specificity. The AUC of the ROC curve for mean attenuation was 0.865 (95% CIs, 0.669–0.968, P < .001) in small and non-calcified pulmonary nodules (Fig 5C).

### Comparison with conventional CT characteristics

In Logistic regression analysis using conventional CT characteristics including diameter and mean attenuation of the lesions, both variables were significant predictors of pulmonary metastases. As with the results using texture features, higher mean attenuation (adjusted odds ratio, 1.007 [95% confidence interval, 1.002–1.011]; P = .003) and larger diameter (adjusted odds ratio, 1.189 [95% confidence interval, 1.022–1.383]; P = .025) were significant differentiators for pulmonary metastases. However, none of conventional CT characteristics showed statistically significant parameter of pulmonary metastases in small non-calcified nodules (P>0.05).

In ROC comparison analysis, mean attenuation measured by texture analysis was a better diagnostic performance than conventional measurement (AUC, 0.958 vs. 0.847; P = .030) (Fig 5A). The diameter of the lesions measured by texture analysis showed a better diagnostic performance than conventional measurement of long diameter (AUC, 0.910 vs. 0.828; P = .032) (Fig 5B). In subgroup analysis, mean attenuation obtained by texture analysis was also a better differentiating performance than conventional measurement (AUC, 0.865 vs. 0.619; P = .049) (Fig 5C).

## Discussion

Chest CT is the most effective method for detecting lung nodules. The development of multidetector CT scanners has enabled the detection of even smaller lung nodules regardless of their nature. Differentiation of pulmonary metastases among pulmonary nodules found on CT is essential at the initial assessment or during follow-up of children with osteosarcoma. Pulmonary nodules found on CT could have variable pathologies, including metastasis, primary pulmonary neoplasm, and intrapulmonary lymph node or benign inflammatory lesions [2, 9, 10]. McCarville et al. showed that 43% of the pulmonary nodules were identified as benign lesions even in pediatric patients with malignant solid tumors [11]. In our study, 43% of all lung nodules proved to be benign nodules. However, the differentiation of malignant and pulmonary benign nodules based on CT morphology is quite difficult. Rosenfield et al. [10] found that tiny malignant pulmonary nodules in children could not be distinguished from benign disease by using CT criteria established in adult patients. McCarville et al. also showed that CT findings such as nodule size and growth and multiplicity of lesions were not reliable in predicting the malignancy of pulmonary nodules in children with extrapulmonary malignant solid tumors [11]. To overcome this difficulty, we introduced a computerized 3D image analysis technique for differentiating pulmonary metastases from non-metastatic nodules in children with osteosarcoma.

In the univariate analysis, many parameters showed a significant difference between metastatic osteosarcomas and non-metastatic lesions. Pulmonary metastases showed higher mean attenuation, standard deviation, variance, effective diameter, surface area, and volume than did non-metastatic pulmonary lesions. We think that the high mean HU, standard deviation, and variance are associated with the presence of calcification or ossification within metastatic pulmonary osteosarcomas. Osteosarcoma is characterized by spindle cells producing an osteoid matrix, and pulmonary metastases in osteosarcoma could manifest as calcified or ossified pulmonary nodules [12, 13]. Effective diameter, surface area, and volume are related to nodule size, and this suggests that the larger the nodule is, the more likely it is to be metastatic. Brader et al. [5] revealed that the presence of calcifications and large nodule size (5 mm or greater) are significantly related with the increased possibility of malignant lesions in children with osteosarcoma. The results of our study are in agreement with those of Brader et al. [5].

Second-order statistics including GLCM ASM, IDM, and entropy also yielded statistically significant results. Entropy, which measures the lack of uniformity within the matrix, was higher in pulmonary metastases. ASM, a measure of the homogeneity of images that have relatively high values for a homogeneous image, was lower in pulmonary metastases, while IDM, which measures the local homogeneity of an image, was higher in pulmonary metastases. The GLCM parameter reflects the inhomogeneity of the nodule, which may be one of the characteristics that distinguishes metastatic lesions from non-metastatic lesions.[14].

Multivariate logistic analysis confirmed that mean attenuation and nodule size were the most significant independent predictors of pulmonary metastases from osteosarcoma. This finding is also consistent with that of a previous report, which found no reliable features among CT characteristics for differentiating metastatic and benign pulmonary nodules, apart from the large size and presence of calcification [5].

In clinical practice, small non-calcified pulmonary nodules are more difficult for radiologists to interpret. The result of our research suggests that the high mean HU obtained from the entire tumor volumes could help differentiate metastatic and benign pulmonary nodules. We believe that this result could be attributed to the high cellular density and the presence of an osteoid matrix in the pulmonary metastatic nodules [15].

The differentiating performance of texture analysis showed superiority over that of conventional assessment in this study. In total group, mean attenuation and lesion diameter measured by conventional method were still significant indicator of pulmonary metastases. However, conventional CT characteristics were no longer effective indicator of pulmonary metastases in small non-calcified nodules. Small nodules of less than 5mm are difficult to measure for accurate attenuation with conventional methods and are likely to include surrounding lung parenchyma. In texture analysis, accurate attenuation of small lung nodules can be measured because it can be analyzed on a pixel basis and pixels showing a particular value can be excluded from the analysis.

Our study has several limitations. First, our research was retrospective and prone to bias. Second, CT examinations were performed using various scanners employing different parameters. This may affect CT attenuation in each voxel and result in the variability of values of texture features. Third, manual segmentation was applied for extracting the texture parameters. Manual segmentation has a problem with reproducibility. However, many other studies have already adopted manual segmentation for identifying the margins of lesions [16]. We applied a nulling technique for reducing personal variability during manual segmentation. Nulling the pixel values of normal lung parenchyma can help identify the boundary of pulmonary nodules and provide reliable values of texture features. Finally, the small sample size is another limitation of our study.

In conclusion, metastatic pulmonary nodules from osteosarcoma could be differentiated from non-metastatic pulmonary lesions by using computerized 3D image analysis. Higher mean attenuation and larger effective diameter were significant predictors for pulmonary metastases, while higher mean attenuation was a significant predictor for small non-calcified pulmonary metastases.

## Supporting information

S1 Appendix Detailed information of the texture features. (DOCX)
