# Supplementary material for: Computerized texture analysis of pulmonary nodules in pediatric patients with osteosarcoma: Differentiation of pulmonary metastases from non-metastatic nodules
Source: PLoS One. 2019 Feb 8;14(2):e0211969. doi: 10.1371/journal.pone.0211969 (PMC6368316; doi:10.1371/journal.pone.0211969)
Supplement: S1 Appendix — (DOCX) [file pone.0211969.s001.docx]

**Supporting information**

**Effective Diameter and Sphericity**

Effective diameter is defined as the diameter of a sphere that has a volume equivalent to that of the segmented pulmonary nodules. Sphericity is a measure of how round an object is. The value of sphericity is defined as the ratio between the volume of a nodule and the volume of a minimum circumscribed sphere (1).

**Discrete Compactness**

The discrete compactness of an object is calculated as the ratio between the contact surface area (Ac) and the theoretical maximum contact surface area (Acmax). The contact surface area is defined as the sum of the surface areas where two adjacent voxels are in common contact. The value of discrete compactness varies from 0 to 1, and the value is related to the circularity of the object. Discrete compactness is calculated using the following equation (2).

**Texture Features from the Gray-level Co-occurrence Matrix**

A gray-level co-occurrence matrix (GLCM) is composed of the element P (i, j), which corresponds to the relative frequency of co-occurrence of the pair of pixels with gray level intensities i and j, separated by a given distance and in a given direction. Various texture parameters derived from the GLCM can be used in image analysis. Five different texture parameters derived from GLCM were used in this study (3).


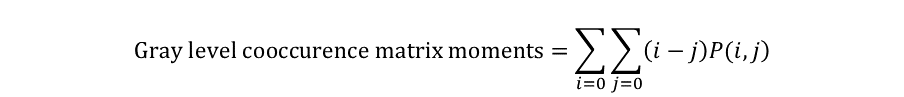
GLCM moments can be defined using the following equation.


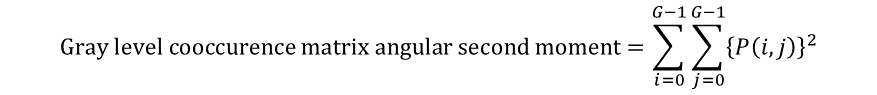
GLCM angular second moment reflects the homogeneity of a given image. The more homogeneous an image is, the higher its GLCM angular second moment.


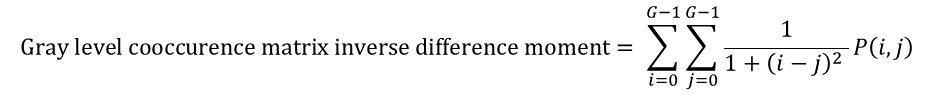
GLCM inverse difference moment is also a measure of the homogeneity of the image. A homogeneous image gives a relatively higher inverse difference moment.

GLCM contrast is a measure of local gray-level intensity variation.


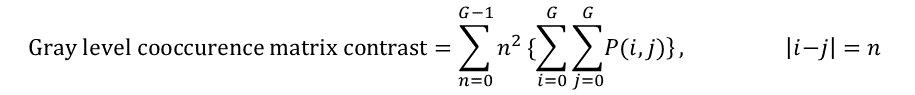


GLCM entropy reflects the homogeneity of an image. A homogeneous image has a high GLCM entropy value.


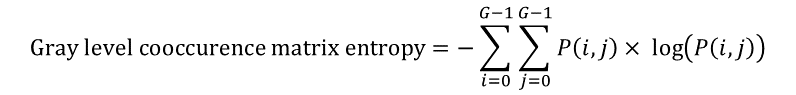


**References**

1. Ye X, Lin X, Dehmeshki J, Slabaugh G, Beddoe G. Shape-based computer-aided detection of lung nodules in thoracic CT images. IEEE transactions on bio-medical engineering. 2009;56(7):1810-20.

2. Bribiesca E. An easy measure of compactness for 2D and 3D shapes. Pattern Recogn. 2008;41(2):543-54.

3. Albregtsen F. Statistical Texture Measures Computed from Gray Level Coocurrence Matrices: Image Processing Laboratory, Department of Informatics, University of Oslo; 2008 [updated November 5, 2008. Available from: <http://www.uio.no/studier/emner/matnat/ifi/INF4300/h08/undervisningsmateriale/glcm.pdf>.
